# Health-related quality of life after laparoscopic repair of giant paraesophageal hernia: how does recurrence in CT scan compare to clinical success?

**DOI:** 10.1186/s12893-020-00772-1

**Published:** 2020-05-20

**Authors:** Henriikka Hietaniemi, Ilkka Ilonen, Tommi Järvinen, Juha Kauppi, Saana Andersson, Harri Sintonen, Jari Räsänen

**Affiliations:** 1 Department of General Thoracic and Esophageal Surgery, Helsinki University Hospital, Haartmaninkatu 4, 00290 Helsinki, Finland; 2 Department of Surgery, Clinicum, University of Helsinki, Helsinki, Finland; 3 Department of Public Health, University of Helsinki, Helsinki, Finland

**Keywords:** Paraesophageal hernia, Laparoscopy, Computerized tomography, Quality of life

## Abstract

**Background:**

Computed tomography (CT) is widely used in the diagnosis of giant paraesophageal hernias (GPEH) but has not been utilised systematically for follow-up. We performed a cross-sectional observational study to assess mid-term outcomes of elective laparoscopic GPEH repair. The primary objective of the study was to evaluate the radiological hernia recurrence rate by CT and to determine its association with current symptoms and quality of life.

**Methods:**

All non-emergent laparoscopic GPEH repairs between 2010 to 2015 were identified from hospital medical records. Each patient was offered non-contrast CT and sent questionnaires for disease-specific symptoms and health-related quality of life.

**Results:**

The inclusion criteria were met by 165 patients (74% female, mean age 67 years). Total recurrence rate was 29.3%. Major recurrent hernia (> 5 cm) was revealed by CT in 4 patients (4.3%). Radiological findings did not correlate with symptom-related quality of life. Perioperative mortality occurred in 1 patient (0.6%). Complications were reported in 27 patients (16.4%).

**Conclusions:**

Successful laparoscopic repair of GPEH requires both expertise and experience. It appears to lead to effective symptom relief with high patient satisfaction. However, small radiological recurrences are common but do not affect postoperative symptom-related patient wellbeing.

## Background

The term giant paraesophageal hernia (GPEH) is used when at least one third of the stomach is situated above the diaphragm. GPEHs constitute approximately 2 to 5% of all paraesophageal hernias [1]. Symptoms caused by GPEH include chest and stomach pain, reflux, heartburn, regurgitation, vomiting, bloating, and shortness of breath. Symptoms can vary from mild or temporary to acute and severe. Emergency surgery is necessary in cases of incarceration, volvulus, and perforation of the stomach [2, 3]. In rare cases, a GPEH can be asymptomatic and discovered incidentally in chest X-rays or computed tomography (CT) scans [4].

The risk of a major medical emergency associated with GPEH is approximately 1% annually and leads to a lifetime risk of approximately 18% at 65 years [5]. The mortality rate after hospitalization for symptomatic GPEH has been reported to be 16.4% for conservative treatment [6], while mortality after emergency operations is higher than after elective surgery [7]. Due to the increased risk and eventual likelihood for emergency surgery, GPEH are commonly treated with elective surgery [6, 8, 9].

The long-term outcomes and durability of laparoscopic repair are debated [10–12]. Complex laparoscopic repair of GPEH has been reported to be safe and successful [13–15] and the postoperative length of stay is shorter and postoperative pain is less severe than after open surgery [11, 16]. On the other hand, radiological evidence of recurrence after laparoscopic surgery is often higher than after open repair [17].

In previous studies, the imaging of choice has usually been barium esophagogram [17, 18]. .However, the barium swallow has become so rare in our clinical practice that few radiologists have experience of executing and interpreting it. Additionally, a barium esophagogram does not reliably show the size of a paraesophageal hernia preoperatively [19] and a sensitivity of only 30% before obesity surgery has been reported [20]. Computed tomography (CT) is currently in common use for both elective and emergency situations and has been used to measure the size of the hiatal orifice with high sensitivity [21].

Health-related quality of life (HRQoL) has been measured individually in previous studies with generic or disease-specific instruments. Minimal correlation between radiological findings and HRQoL has been shown [14, 18, 22].

The objective of this study was to evaluate the treatment outcomes of elective laparoscopic GPEH repair in a single tertiary care centre. The primary outcome was radiological recurrence in CT and its association with current symptoms and HRQoL. We also evaluated possible pre- and perioperative factors that could be linked to unsatisfactory outcomes or GPEH recurrence.

## Methods

This was a consecutive case-series study on laparoscopic GPEH repair with follow up for radiological surveillance of hernia recurrence and patient postoperative HRQoL.

### Patient cohort

The patient cohort included patients diagnosed with GPEH and subsequently operated electively with laparoscopy for GPEH in a single tertiary care hospital between 2010 and 2015. Patient information was collected retrospectively from electronic medical records (EMR) and included date of operation, preoperative symptoms, comorbidities, smoking, body mass index (BMI), preoperative proton pump inhibitor (PPI) use, pre- and postoperative laboratory and radiological tests, length of hospital stay, complications, reoperations, mortality, readmissions, intensive care unit stay, duration of operation, urgency of operation, possible additional procedures, referring hospital, operating surgeon, and follow-up data. The size of the hernia was based primarily on the original radiologist’s report of preoperative CT, or if unavailable, the assessment in the operation report or preoperative gastroscopy.

### Surgical technique

The operations were performed laparoscopically using five ports with a Nathanson liver retractor. During the operation, the hernia sac is dissected circumferentially and reduced from the mediastinum. The esophagus is mobilised to obtain > 2 cm of tension-free esophagus beneath the diaphragm. A hiatoplasty is performed with sutures and the diaphragmatic crura are reinforced with mesh if needed. A fundoplication is commonly performed, predominantly a floppy Nissen fundoplication with a thick nasogastric tube as a bougie. In patients not suitable for fundoplication, a simple gastropexy is performed. The operation reports were reviewed to obtain more detailed information about the surgery. We examined the reports for possible fundoplication, use of mesh reinforcement, lengthening of esophagus, and other features.

### Quality of life

The identified patients were sent an invitation to participate in the study with a letter of information, study consent, and questionnaires for generic 15D HRQoL and disease-specific Gastroesophageal Reflux Disease-Health Related Quality of Life (GERD-HRQL) instruments. The 15D is a generic, 15-dimensional self-administered quality of life instrument [23]. The 15D score was obtained from the patients that reported scores for each of the 15 dimensions. These scores were compared to the scores from the general population from the Terveys 2011 health study [24]. The GERD-HRQL was developed to measure symptom severity related to gastroesophageal reflux disease but was used here as similar symptoms are reported with GPEH [25, 26]. The questionnaires were administered only postoperatively. The patients were divided into groups of symptom severity by their GERD-HRQL scores. A score of 0–5 was considered excellent, 6–10 good, 11–15 fair, and 16 and over, poor.

### Radiological recurrence

The patients who consented to participate were offered a non-contrast CT scan of the chest and upper abdomen. A radiologist’s report was received to determine the recurrence of the hernia. A recurrent hernia was defined as a hernia with > 2 cm of the stomach above the diaphragm. The hernia was considered small, or minor recurrence, if it was 2 to 5 cm and a large recurrent hernia, or major recurrence, was > 5 cm, as defined in prior studies [18, 22, 27].

### Statistical analysis

We analysed results with statistical tests using IBM SPSS Statistics Version 24. Chi-squared or Fisher’s exact test were used between two categorical variables. The Mann-Whitney test was used for analyses with interval independent variables and categorical dependent variables. The Kruskal-Wallis test was used for analyses with interval independent variables and ordinal dependent variables. Spearman correlation was used for analyses with two interval variables. A simple logistic regression was used for the analyses with categorical independent variables and interval dependent variables. Results were considered statistically significant when the two-tailed *p*-value was < 0.05.

### Ethics approval

The study was submitted to and approved by the Research Ethics Committee of the Faculty of Medicine of Helsinki University (code 419/13/02/02/2015) and by the Institutional Review Board (IRB) of the Helsinki University Hospital Heart and Lung Centre (decision 8/2016).

## Results

**Fig. 1:**
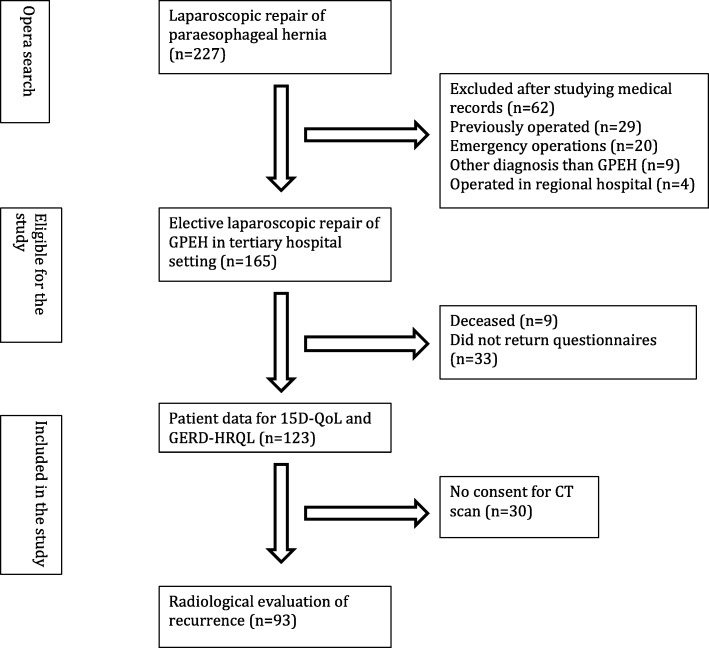
Patient flowchart. The figure follows the steps we took to include the patients in our study. GPEH = Giant paraesophageal hernia. 15D QoL = 15 dimensional quality of life tool. GERD-HRQL = Gastroesophageal reflux disease-health related quality of life

Patient demographics are presented in Table 1 and the patient flow in Fig. 1*.* A total of 227 patients underwent laparoscopic repair of paraesophageal hernia between 2010 and 2015. We excluded open procedures, both planned and converted, patients who had been previously operated on (*n* = 29), emergency operations (*n* = 20), patients with non-giant paraesophageal hernia (*n* = 9) and 4 patients who had been operated at a regional hospital. We included 165 patients in our study. The total number available for QoL analysis was 123 and a total of 93 for radiological analysis. The median follow-up time from the operation to receiving the questionnaires was 33 months (range 10 to 72 months).

**Table 1:** Patient demographics

	n (%)
Gender	
Male	43 (26.1)
Female	122 (73.9)
Age (years)	
< 50	6 (3.6)
51–60	35 (21.2)
61–70	64 (38.8)
≥ 70	60 (36.4)
Age-adjusted CCI ^a^	
0–2	71 (43.0)
≥ 3	94 (57.0)
Pulmonary disease ^b^	36 (21.8)
History of ever smoking	42 (25.5)
Preoperative BMI > 35 kg/m^2 c^	12 (9.0)
Anemia	111 (67.3)
Symptomatic	158 (95.8)
Hernia size	
30–49%	26 (16.6)
50–74%	77 (46.7)
75–99%	22 (13.3)
100%	32 (19.4)
Hernia class	
III	148 (89.7)
IV	17 (10.3)
Fundoplication	149 (90.3)
Crural mesh	8 (4.8)
Length of stay (days)	
1–2	66 (40.2)
3–4	51 (30.9)
5–7	26 (15.8)
≥ 7	21 (13.1)

^a^*CCI* Charlson comorbidity index

^b^Asthma or chronic obstructive pulmonary disease (COPD)

^c^Body Mass Index (BMI), data was available for 133 patients

### Surgical features

Most operations (*n* = 134, 81.2%) were performed by one surgeon (JR) and altogether five different surgeons performed these operations. Mesh reinforcement was used in 8 patients (4.2%) and absorbable mesh was used in all except one of them. Esophageal lengthening was considered necessary after mobilization in none of the patients. A fundoplication was performed in 149 patients (90.3%). The mean duration of operation was 125 min (SD ± 51, range 51–348 min). Robot-assisted surgery was used for 9 patients (5.5%).

### Adverse events

Complications after laparoscopic operation were reported in 27 patients (16.4%); 4 patients had more than one complication. The complications were classified according to the Clavien-Dindo classification [28, 29]. There were 18 patients (10.9%) with grade-II complications with a median Charlson comorbidity index (CCI) of 1 [30]. A grade-III complication was reported in 7 patients (4.2%) with a median CCI of also 1. One patient (0.6%) had a grade-IV complication and one patient (0.6%) had a grade-V complication with CCIs of 2 and 4, respectively. Complications are summarised in Table 2.

**Table 2:** Complications by Clavien-Dindo classification

Complications	n (%)
Grade II	18 (10.9)
Wound infection	6 (3.6)
Other infection	3 (1.8)
Lung embolism	3 (1.8)
Exacerbation of pulmonary disease	2 (1.2)
Urinary retention	2 (1.2)
Atrial fibrillation	1 (0.6)
Partial infarction of the spleen	1 (0.6)
Grade III	7 (4.2)
Chylothorax	1 (0.6)
Esophageal stricture	1 (0.6)
GE-junction perforation	1 (0.6)
Small intestine perforation	1 (0.6)
Small intestine strangulation	1 (0.6)
Gastric paralysis	1 (0.6)
Gastric strangulation	1 (0.6)
Grade IV	1 (0.6)
Gastric perforation	1 (0.6)
Grade V	1 (0.6)
Perforation of the duodenum	1 (0.6)

Nine deaths occurred during follow up. There was one postoperative death within 30 days. This patient was considered high risk preoperatively, with an age-adjusted CCI of 6. According to autopsy, death was due to cryoglobulinemic vasculitis which caused intestinal perforation. The other eight deaths were not directly related to GPEH and occurred a mean of 22 months (SD ± 14.6) after operation.

In total, 16 patients (9.7%) required reoperation. Of these, 10 (6.1%) occurred within 30 days of the primary operation, with reasons including recurrent hernia (*n* = 3), gastric paralysis (*n* = 2), small intestine strangulation (*n* = 1), suspected bleeding (*n* = 1), gastric perforation (*n* = 1), small intestine perforation (*n* = 1), and leakage at the GE junction (*n* = 1). The reoperations were carried out mainly using open technique, either laparotomy or thoracotomy. The patient with suspected bleeding was reoperated laparoscopically and for two patients endoscopic intervention with PEG was sufficient. The causes for a later reoperation were hernia recurrence and in one case gastric strangulation.

The median hospital stay postoperatively was 3 days (range 1 to 34 days).

### Recurrence and patient reported outcomes

Of the 165 operated patients, 158 (95.8%) were symptomatic preoperatively. Disease-specific pre- and postoperative symptoms are presented in Table 3. The scores derived from the GERD-HRQL questionnaire were mainly excellent (66%) or good (12%). A fair score was achieved by 12 patients (10%) and a poor score by 15 patients (13%). The median GERD-HRQL score was 2 (range 0 to 56).

**Table 3:** Patient-reported symptoms pre- and postoperatively based on electronic medical records and current information obtained

Symptom	*n* = 162	preoperative n (%)	postoperative n (%)
Pain		94 (57.0)	13 (8.0)
Heartburn		40 (24.2)	3 (1.9)
Regurgitation		31 (18.8)	2 (1.2)
Vomiting		37 (22.4)	0 (0)
Dysphagia		49 (29.7)	13 (8.0)
Difficulty swallowing			
	solid	35 (21.2)	0 (0)
	soft	2 (1.2)	0 (0)
	liquid	2 (1.2)	0 (0)
Dyspnea		25 (15.2)	2 (1.2)
Bloating		1 (0.6)	9 (5.6)
Early satiety		30 (18.2)	0 (0)
Aspiration		9 (5.5)	0 (0)
Cough		10 (6.1)	0 (0)
PPI ^a^		97 (58.8)	16 (9.9)

^a^Daily use of proton pump inhibitors

A total of 118 patients (71.5%) answered the question regarding current overall satisfaction. Seven (5.9%) patients reported overall dissatisfaction for symptom control postoperatively. The GERD score correlated with satisfaction (*p* = 0.001). The unsatisfied patients had a median score of 19 (range 6 to 54) and the satisfied patients had a median score of 1 (range 0 to 19).

**Fig. 2:**
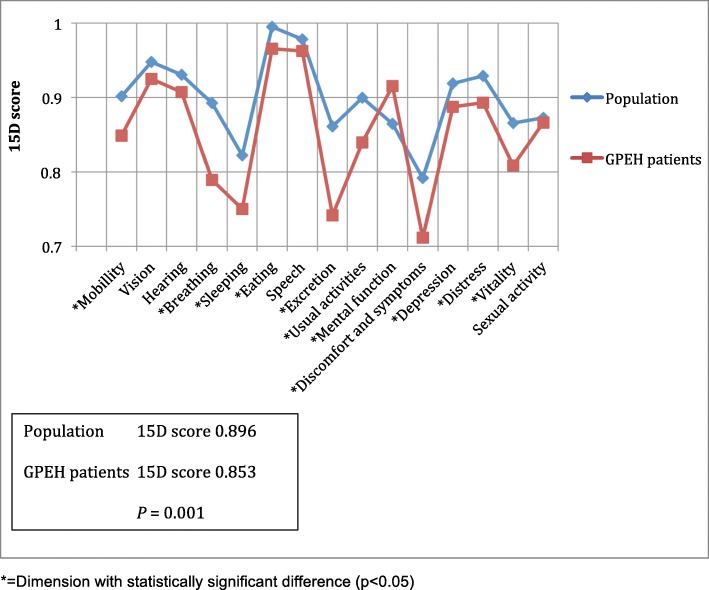
15D Quality of life score of patients with laparoscopically repaired giant paraesophageal hernia compared to the general population. The graph displays the scores for the 15 different dimensions and the total score for quality of life in our patients and the general population. GPEH = Giant paraesophageal hernia

The 15D quality of life survey was returned by 121 patients (73.3%). The results are presented in Fig. 2. The overall 15D HRQoL score was close to the age-adjusted average. The mean 15D score was 0.85 for patients and 0.90 for the general population (*p* = 0.001). The mean 15D total score was slightly better in the group with radiological recurrence (0.89) than in patients without recurrence (0.84) (*p* = 0.03). The only dimension with a statistically significant difference between patients with and without recurrence was in the bladder and bowel functions (excretion) (*p* = 0.012).

**Fig. 3:**
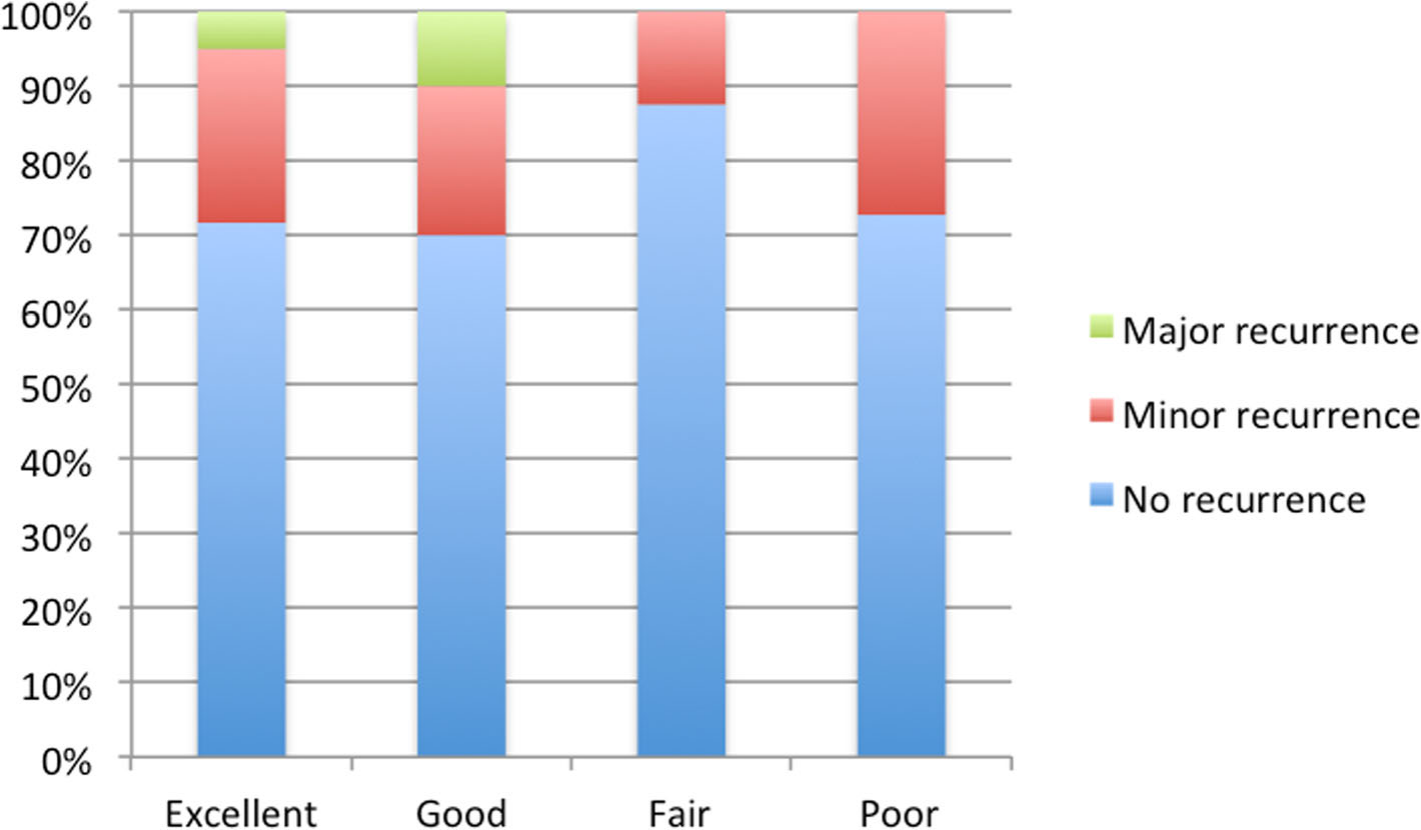
Symptom-related quality of life and recurrence. The graph shows the percentage of patients with recurrent paraesophageal hernia, diagnosed by computerized tomography, in different groups of symptom severity

A current CT scan was obtained from 92 patients (59% of operated patients). The median follow-up period from operation to CT was 39 months (range 12–79). Major recurrence, as defined in the Methods section, was revealed by CT in 4 patients (4.3%). The total recurrence rate in our patients, including the patients reoperated for recurrence preceding our follow up, was 29.3%. The symptoms evaluated by overall GERD-HRQL scores did not correlate with radiological findings (*p* = 0.124) (Fig. 3).

### Risk factors

Known preoperative pulmonary disease was associated with postoperative complications (*p* = 0.036), a poorer quality of life in the 15D score (*p* = 0.011) and greater symptoms in GERD-HRQL (*p* = 0.002). Older age and longer operating time correlated with a prolonged length of stay (both *p* = 0.001). We did not identify any factors from patient or operation characteristics that could predict hernia recurrence.

## Discussion

The most important finding of this study is that laparoscopic repair of GPEH in a specialised tertiary referral centre is safe, has a low recurrence rate for large hernia, and has good long-term patient satisfaction as measured by generic HRQoL and disease-specific symptom questionnaires. However, there is a moderate risk for grade I or II complications and overall hernia recurrence.

We observed a considerable rate of radiological recurrence after laparoscopic GPEH repair. Recurrent hernia occurred in 29% of the patients. However, only 4 patients (4.3%) had major recurrent hernia, which may still be susceptible to the risks associated with giant paraesophageal hernia, most notably incarceration, volvulus and perforation of the stomach. Recurrent hernia does not seem to affect patient HRQoL, which may explain the minimal reported need for reoperations in the literature for recurrent hernia (approximately 0.5 to 4.4%) even though radiological recurrence is common [18, 31, 32].

In initial studies, the radiological recurrence rate for GPEH was often higher in laparoscopic than in open procedures, with laparoscopic recurrence rates from 13% up to 42% [17]. Despite this early outcome report, laparoscopic operations have become the predominant operative approach [33]. As surgeons have improved laparoscopic operation techniques, results have improved over time and experience. The recurrence rates of laparoscopic repair of GPEH in retrospective series ranged from 12 to 21% [11, 34]. However, in a recent prospective series the rate was as high as 32.7% at 1 year. [19] The total recurrence rate of 29.3% in our series is similar.

Our relatively high recurrence rate may depend on several factors. First, we performed follow up with CT, which may be more sensitive to detect recurrent hernias than a barium swallow test, especially true paraesophageal and sliding hernias. Second, we did not perform Collis gastroplasty, which may increase the number of radiological recurrences. We avoided lengthening procedures and mesh reinforcement to reduce additional risks. These include leakage and later stricture formation at the site of gastroplasty and erosion, stenosis, and dysphagia caused by mesh. The value of recurrence prevention by the most commonly currently used biological meshes in long-term follow up has been questioned [35]. As our patients had good HRQoL even after minor radiological hernia, it is unclear if better results would have been achieved with additional procedures [36].

In our data, the HRQoL as defined by the 15D score was close to the population average. A slight inferiority was detected in breathing, and bladder and bowel functions. The reasons for this result remain unclear and further examination would require a much larger series. The 15D score was slightly higher in the group of patients with recurrent hernia. While this group had fewer problems in bladder and bowel functions, the association with GPEH is uncertain. The preoperative quality of life was not measured here, but overall HRQoL improves with a laparoscopic GPEH repair [22].

Symptom relief (as measured by the GERD-HRQL score) after laparoscopic GPEH repair was good in our series. This has also been shown in previous studies, with good to excellent results reported by 77 to 90% of patients [17, 18, 32]. We also observed that as in many previous studies [17, 18] the radiological findings did not correlate with clinical symptoms.

Perioperative mortality was low (0.6%), similar to the 0 to 1.6% previously reported. The 30-day reoperation rate was 6.1%. The complication rate (16.4%) was comparable to the rates reported in previous studies (9 to 19%). Most complications were minor (Clavien-Dindo grade II), with wound infections being the most common. There were some cases of leakage and perforation, which is comparable to other series [17, 18, 31].

The strength of our study was that we reached a high percentage of our patients for follow-up surveys and CT scans. This study also has some limitations. This was a retrospective cross-sectional study with a single timepoint surveillance for imaging joined with HRQoL questionnaires. In addition, this study was limited to laparoscopic operations that were successfully completed; conversions to open repair were excluded. Thus, we did not use an intention-to-treat analysis, but rather concentrated on the end results of laparoscopy. We also acknowledge that there were no structured questionnaires for both HRQoL and symptoms preoperatively, which could cause recall bias in the presentation of symptoms. Despite a significant portion of patients willing to participate in this study, some patients declined an additional follow-up CT scan, which could cause selection bias in the results.

## Conclusions

In conclusion, although laparoscopic repair of GPEH is a complex procedure, when executed by an experienced surgeon it appears to lead to effective symptom relief with high patient satisfaction. Minor radiological recurrence is common but does not affect HRQoL or satisfaction. These findings suggest that the laparoscopic approach is a feasible first-line surgical strategy for GPEH repair. Further research with longer follow-up times and wider, population-based studies are required to determine the optimal approach to repair GPEH.

## Data Availability

The datasets generated during and/or analysed during the current study are available from the corresponding author on reasonable request.

## Abbreviations

BMI: Body Mass Index
CCI: Charlson Comorbidity Index
COPD: Chronic obstructive pulmonary disease
CT: Computed tomography
EMR: Electronic medical records
GERD-HRQL: Gastroesophageal reflux disease –health related quality of life
GPEH: Giant paraesophageal hernia
HRQoL: Health-related quality of life
IRB: Institutional review board
PPI: Proton pump inhibitor
